# A Conceptual Reference Architecture for Robust, Leakage-Resilient and Verifiable Access Control in Secure IoT Outsourcing

**DOI:** 10.3390/s26154878

**Published:** 2026-08-02

**Authors:** Siddig M. Elkhider

**Affiliations:** Department of Computer Engineering, College of Computer Sciences and Information Technology, King Faisal University, Al-Ahsa 31982, Saudi Arabia; selnaiem@kfu.edu.sa

**Keywords:** IoT security, attribute-based encryption, multi-authority key management, blockchain logging, proxy re-encryption, leakage resilience, post-quantum cryptography, federated anomaly detection

## Abstract

Outsourcing Internet-of-Things (IoT) data and computation to cloud and fog infrastructure exposes both the data and the access-control process to integrity, confidentiality, and privacy risks. Attribute-based encryption (ABE) provides fine-grained access control but, as deployed today, suffers from single-authority bottlenecks, expensive policy updates, weak auditability, and exposure to secret-key leakage, classical primitives are additionally threatened by future quantum adversaries. This paper does not propose a new cryptographic scheme. Instead, it contributes a conceptual reference architecture that systematizes how a set of existing, standardized primitives can be composed into a single access-control framework for IoT outsourcing, and it makes the resulting design precise enough to reason about. Concretely, we (i) define a system model and a threat model covering passive, active, colluding, bounded-leakage, and harvest-now-decrypt-later quantum adversaries; (ii) instantiate each layer with a named construction decentralized multi-authority ABE, attribute-based proxy re-encryption for policy updates, a bounded leakage resilient key model, ASCON lightweight AEAD, and ML-KEM/ML-DSA post-quantum primitives, together with a permissioned, on-chain digest/off-chain payload logging layer; (iii) specify the end-to-end data flow and module interfaces; and (iv) give a goal-by-goal security rationale and an analytical evaluation based only on standardized parameter sizes and asymptotic complexity. We are explicit about what is inherited from prior work, what remains to be proven for the composed system, and that a measured prototype evaluation remains future work. The intended value of this paper is to provide a clear, composable, and honestly scoped design that subsequent implementation studies can build upon.

## 1. Introduction

The growth of IoT has produced unprecedented connectivity across domains from smart healthcare to industrial automation [1,2]. Because IoT endpoints are constrained in computation, memory, and energy, both storage and computation are frequently outsourced to cloud and fog providers. Outsourcing, however, moves sensitive data and the enforcement of access policies into partially trusted infrastructure, raising risks to data confidentiality, policy integrity, and third-party information leakage [1].

Attribute-based encryption, particularly ciphertext-policy ABE (CP-ABE) [3,4,5], lets a data owner bind ciphertexts to an access structure so that only users whose attributes satisfy the policy can decrypt them, without identifying individual recipients. Despite substantial progress, four limitations persist for IoT outsourcing. First, the common single trusted authority is both a scalability bottleneck and a single point of failure. Second, policy updates typically require re-encrypting data, which is prohibitive at the IoT scale. Third, ABE alone provides no verifiable audit trail, hindering accountability and forensics. Fourth, real deployments leak partial key information through side channels [6], and classical public-key primitives will be broken by a sufficiently capable quantum computer via Shor’s algorithm [7].

A natural response is to combine complementary technologies with verifiable logging, decentralized key management, efficient policy updates, proactive anomaly detection, and quantum-resistant primitives. The difficulty is that “combining” is easy to assert and hard to make precise: Without a shared threat model, named constructions, and an explicit data flow, such a combination cannot be reasoned about or reproduced. The contribution of this paper is to supply exactly that precision.

### Contributions

This paper is a conceptual architecture and systematization article. Our contributions are as follows:

- A system and threat model (Section 3) that states the entities, trust assumptions, adversary classes, and security goals for an outsourced IoT access-control system so that subsequent claims can be evaluated against a fixed model.
- A reference architecture and end-to-end data flow (Section 4) that specifies how the modules compose, where the trust boundaries lie, and what each interface exchanges, organized around five explicit architectural principles (policy–payload separation, digest-on-chain, gid-anchored binding, version-tag atomicity, and key-domain separation) together with a precise entity-state and version-synchronization specifications, which distinguish the design from a survey-style stack of technologies.
- A concrete, design-level instantiation of each layer with a named, citable construction (Section 5): decentralized MA-ABE, attribute-based proxy re-encryption for policy update, a bounded-leakage key model, ASCON, ML-KEM, and ML-DSA; and a permissioned, on/off-chain logging design.
- A goal-by-goal security rationale (Section 6) with proof sketches that attribute each guarantee its underlying result and an honest account of what remains unproven for the composed system.
- An analytical evaluation (Section 7) using only standardized parameter sizes and asymptotic complexity, plus a qualitative design-space comparison that is explicitly not a measured benchmark.

We emphasize at the outset what this paper does *not* do: It does not introduce a new cryptographic scheme, and it does not report measured performance. These are deliberate scope decisions discussed in Section 9.

The remainder of this paper is organized as follows. Section 2 reviews related work. Section 3 defines the system and threat model. Section 4 presents the reference architecture and data flow. Section 5 details the component constructions. Section 6 gives the security analysis. Section 7 presents the analytical evaluation. Section 8 discusses trade-offs, Section 9 states limitations and future work, and Section 10 provides conclusions.

## 2. Related Work

### 2.1. ABE and Outsourced Decryption

CP-ABE [5] encrypts under an access structure and is well suited to fine-grained sharing. To fit constrained devices, outsourced/partial decryption delegates the heavy pairing work to a proxy while preserving security [8]. These techniques reduce on-device costs but do not, by themselves, remove the single-authority bottleneck, provide auditability, or resist quantum attack.

### 2.2. Leakage Resilience

Practical implementations leak partial information about secret keys through power, timing, or electromagnetic side channels. Leakage-resilient cryptography models this explicitly: In the bounded-leakage model [6] the adversary may obtain up to λ bits of leakage on secret state, while the continual-leakage model permits bounded leakage per time period across key refreshes. Our framework adopts a bounded-leakage model for attribute and session keys and builds on the dual-system leakage-resilient ABE of Lewko, Rouselakis, and Waters [9], which we adopt as the named instantiation (Section 5.4).

### 2.3. Multi-Authority ABE

Decentralized MA-ABE removes the central authority: Independent authorities issue keys for disjoint attribute sets using only shared global parameters and a global user identifier, with collusion resistance enforced through that identifier [10,11]. This directly addresses scalability and single-point-of-failure concerns.

### 2.4. Policy Update via Proxy Re-Encryption

Proxy re-encryption (PRE) [12] lets a semi-trusted proxy transform a ciphertext from one key (or policy) to another without learning the plaintext. Attribute-based PRE applies this to ABE, enabling policy changes without full data re-encryption, which is the property we require for dynamic IoT policies.

### 2.5. Blockchain Logging

Permissioned ledgers such as Hyperledger Fabric [13], using Byzantine-fault-tolerant ordering [14], provide tamper-evident, non-repudiable logs suitable for closed sets of known operators, which are typical in outsourcing settings, in contrast to a bare append-only hash chain.

### 2.6. Federated Learning and Robust Aggregation

Federated learning [15] trains a shared model from decentralized data without centralizing raw records, and it is attractive for privacy-preserving IoT intrusion/anomaly detection. Robustness and privacy require additional mechanisms: secure aggregation [16], differential privacy, Byzantine-robust aggregation [17,18], and non-IID handling [19].

### 2.7. Lightweight and Post-Quantum Cryptography

ASCON is the NIST lightweight-cryptography standard for constrained authenticated encryption [20]. For quantum resistance, NIST has standardized ML-KEM (FIPS 203, from CRYSTALS-Kyber [21]) for key encapsulation and ML-DSA (FIPS 204, from CRYSTALS-Dilithium [22]) for signatures. Pairing-based ABE is itself *not* post-quantum; lattice-based ABE is the forward-looking path, which we treat as future work rather than a present guarantee.

The novelty we claim is not in any of these primitives individually but in a precise, threat-modeled composition of them for the IoT-outsourcing setting, together with an honest analysis of what the composition does and does not guarantee.

## 3. System Model and Threat Model

### 3.1. Entities

- Data Owner (DO). Defines access policies, encrypts data, derives re-encryption keys for policy updates, and signs policy-update requests.
- IoT Devices. Constrained producers/consumers that perform lightweight symmetric encryption and outsourced (partial) decryption.
- Attribute Authorities (AAs). A set $\left\{{\mathrm{AA}}_{1},\ldots ,{\mathrm{AA}}_{n}\right\}$, each managing a disjoint attribute universe and issuing attribute keys.
- Cloud/Fog Provider and Proxy. Stores ciphertexts, performs outsourced decryption, and applies proxy re-encryption on policy update.
- Permissioned Ledger. Endorsing peers and an ordering service recording verifiable, non-repudiable access events.
- Federated Monitor. An aggregator and participating clients that train an anomaly-detection model over decentralized access telemetry.
- Data Users. Request access, granted if their attributes satisfy the current policy.

### 3.2. Trust Assumptions

The cloud/fog provider and the proxy are honest-but-curious: They follow the protocol but try to learn plaintext or policy details. Attribute authorities are independent, and the adversary may statically corrupt a subset of size at most t<n. The ledger’s ordering service is correct under a Byzantine threshold (fewer than one-third faulty orderers for PBFT-style ordering; only crash faults for Raft). IoT devices may leak a bounded amount of key material through side channels. Cryptographic hash functions are collision-resistant. We make the relationship between these assumptions and the active adversary of Section 3.3 explicit. The honest-but-curious assumption is the baseline under which the confidentiality goals (G1–G4) are argued. The integrity-oriented goals are deliberately designed not to depend on it: Log integrity (G5) must hold even if the cloud later turns malicious and attempts tampering (which is precisely why event digests are signed and anchored on a BFT-ordered ledger rather than trusted to the cloud), and correctness of outsourced decryption (G7, below) must hold against a malicious or faulty proxy via verifiable outsourced decryption. Thus the active adversary of Section 3.3 models both external attackers and the failure of the honest-but-curious assumption for integrity properties, only the confidentiality-of-policy-content arguments (G4) rely on the proxy remaining semi-honest.

### 3.3. Adversary Model

We consider a probabilistic polynomial-time adversary A with the following, possibly combined, capabilities:

- Passive: observes ciphertexts, public keys, and ledger entries.
- Active: attempts to tamper with stored data, logs, or messages, including a proxy that returns incorrect partial-decryption results.
- Colluding Users: pool attribute keys to satisfy a policy none of them satisfies individually.
- Corrupt Authorities: controls up to *t* AAs and their issued keys.
- Bounded Leakage: adaptively queries a leakage function *f* on ABE attribute-key state, learning at most λ bits per key (Definition 1).
- Quantum (Harvest Now Decrypt Later): records traffic now and, with a future cryptographically relevant quantum computer, runs Shor’s algorithm [7] to break classical key exchange/signatures.

**Definition 1** 
(Bounded Leakage)**.** *A key-handling scheme is λ-leakage-resilient if, for every PPT adversary that adaptively obtains $f_{i}\left(\mathrm{sk}\right)$ with $\sum_{i}\left|f_{i}\left(\mathrm{sk}\right)\right|\leq \lambda$, the scheme’s target security game remains hard. In this architecture, the model applies to ABE attribute keys, instantiated by the dual-system leakage-resilient ABE of Lewko, Rouselakis, and Waters [9], in which a key of n group elements over a group of prime order p tolerates leakage up to $\lambda \approx \left(n-1-2c\right)\log_{2}p$ bits for a small constant c; thus, λ is a tunable constant fraction of the key length, and raising the leakage budget enlarges keys proportionally ($n=\Theta (\lambda /\log_{2}p)$). Transport/session keys are not covered by this model and are protected instead by containment (Section 5.4).*

### 3.4. Security Goals

G1 Confidentiality: This ensures IND-CPA security of payloads under the access policy. G2 Collusion Resistance: Colluding users and up to *t* corrupt AAs cannot carry out decryption beyond their combined legitimate rights. G3 Leakage Resilience (Attribute Keys): G1–G2 hold under λ-bounded leakage on attribute keys. G4 Update Privacy: Policy updates reveal neither plaintext nor (when policy-hiding is used) the new policy to the proxy. G5 Log Integrity/Non-Repudiation: Recorded access events are tamper-evident and attributable. G6 Post-Quantum Confidentiality of Transport: Session keys established for outsourced data resist a future quantum adversary. G7 Verifiable Outsourced Decryption: A user can detect an incorrect partial-decryption result returned by a malicious or faulty proxy.

## 4. Reference Architecture and Data Flow

**Figure 1:**
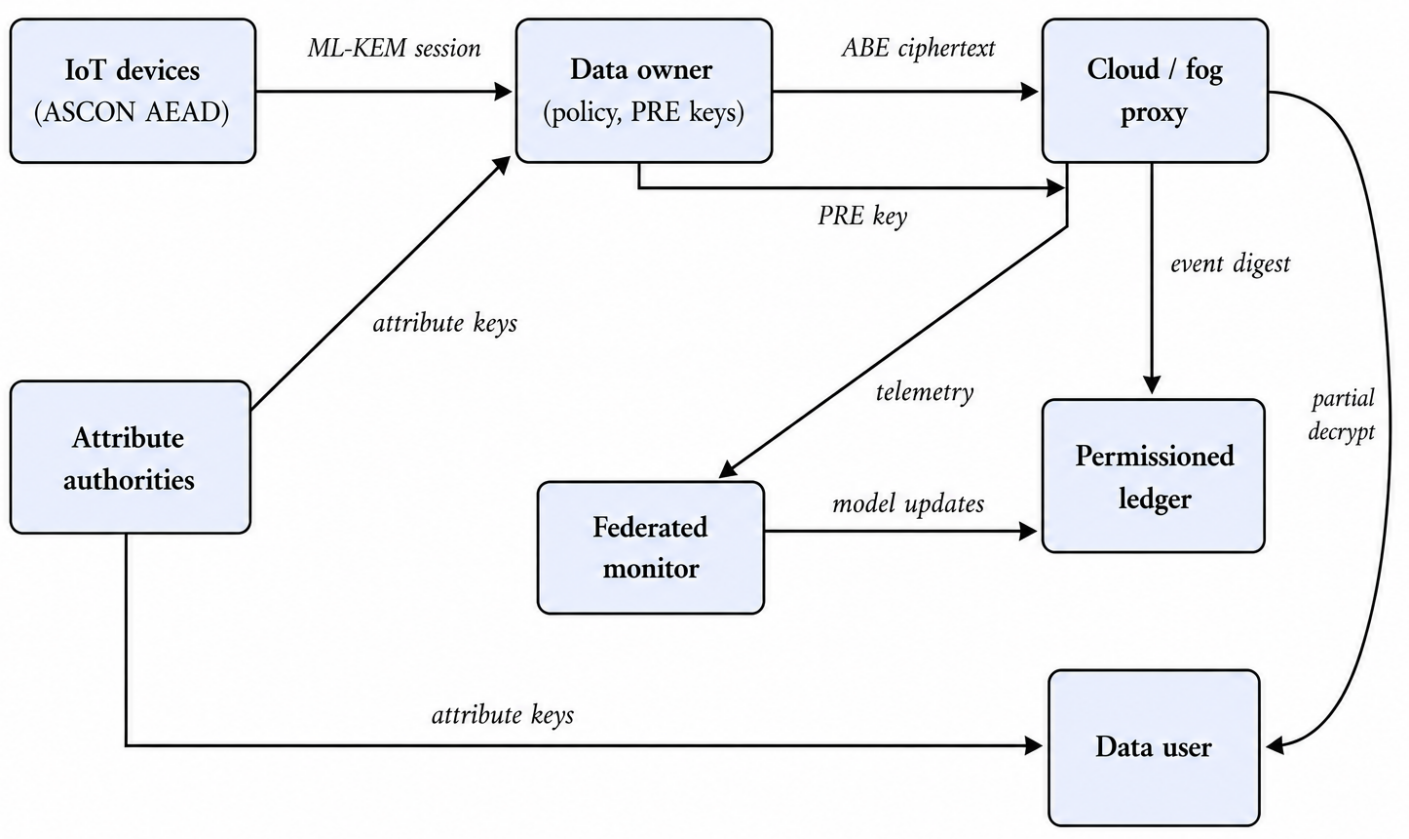
Reference architecture. Arrows denote the principal data and key flows. ML-DSA signatures (omitted for clarity) authenticate ledger entries and policy-update requests.

Figure 1 shows the reference architecture. The layers are orthogonal in function but share a single trust model: The lightweight/PQC layer protects transport and device data, MA-ABE enforces fine-grained access, PRE handles policy change at the proxy, the ledger records every decision, and the federated monitor watches access telemetry for anomalies.

### 4.1. End-to-End Data Flow

1. Enrollment. Global parameters are published, each AA runs its authority setup. A user obtains attribute keys from the relevant AAs under a global identifier that binds the keys together for collusion resistance.
2. Encryption/Outsourcing. The DO encrypts the payload with a symmetric key using ASCON, encapsulates that key under the data user population via MA-ABE (with the symmetric key carried in the ABE-protected header), and uploads the ciphertext to the cloud. Device-to-cloud sessions use ML-KEM-established keys.
3. Access. A user submits an access request, and the cloud performs outsourced (partial) decryption if the user’s attribute keys satisfy the policy, returning a partially decrypted token that the user finalizes locally.
4. Logging. The cloud writes a signed digest of the event (requester, resource, decision, timestamp, and payload hash) to the ledger, the payload/log body is stored off-chain under a Merkle commitment.
5. Policy Update. To change a policy, the DO derives a re-encryption key from the old and new access structures and sends it to the proxy, which transforms the policy-bound ciphertext components in place, the symmetric payload is untouched.
6. Monitoring. Access telemetry feeds the federated monitor, for which its clients train locally and submit robust, privacy-preserving updates, and detected anomalies are themselves logged.

### 4.2. Architectural Principles

What distinguishes this architecture from an ad hoc security stack is a small set of composition rules that every module must respect; they are this paper’s organizing design contribution and are referenced throughout.

- P1—Policy–Payload Separation. Payloads are encrypted once under a symmetric AEAD key, and all access-control operations (encryption under a policy, PRE update, and revocation) act only on the small ABE-protected header. No policy operation ever touches payload bytes, which is what makes update and revocation costs independent of data size.
- P2—Digest-on-Chain, Body-off-Chain. Only constant-size authenticated digests are written to the ledger; bodies live off-chain under Merkle commitments. Auditability never scales with payload volume.
- P3—Gid-Anchored Key Binding. Every key share issued by any authority is bound to the user’s global identifier, so cross-user and cross-authority combinations are non-functional by construction.
- P4—Version-Tag Atomicity. Every ciphertext header and attribute key carries a monotonic version tag; policy updates and revocations take effect as a single version-tag swap, giving well-defined semantics under concurrency (Section 4.3).
- P5—Key-Domain Separation. Transport keys (ML-KEM-derived), payload keys (ASCON), attribute keys (MA-ABE), and signing keys (ML-DSA) belong to disjoint domains: No key is reused across modules, and no module’s secret state is input to another module. This is the structural assumption that makes component-wise security arguments meaningful (Section Compositional Security: Assumptions, Invariants, and Ideal Functionalities).

### 4.3. Entity State and Version Synchronization

We make the runtime behaviour precise. Each entity maintains the following states: DO: for each data object, the current access structure A and its policy version $v_{P}$; each AA_*i*_: for each attribute it manages, an attribute version $v_{a}$ and the set of issued key shares; proxy/cloud: stored ciphertext headers tagged with $v_{P}$ and re-encryption keys indexed by (object, $v_{P}\to v_{P}+1$); ledger: append-only, totally ordered event digests, including authenticated version records announcing new $v_{P}$/$v_{a}$ values; user: key shares tagged with the $v_{a}$ under which they were issued.

Initiation. Policy updates are initiated only by the DO (the federated monitor may recommend an update by raising an anomaly event, but it cannot change policy). The DO signs the update request with ML-DSA, and the proxy applies it and logs the version record.

Synchronization. Version counters are monotonic and are published as signed version records on the ledger, which serves as the single ordering authority (P2). An AA learns of a revocation-relevant update from the ledger and issues re-versioned key shares to still-authorized users; users refresh lazily on their next access attempt (a decryption against a newer $v_{P}$ fails closed and triggers a key-refresh request).

Concurrency Semantics. An access request is evaluated against the ciphertext version current at admission: The proxy atomically reads the header’s $v_{P}$ once, when the request is logged. The PRE transform of a header is a single compare and swap on the version tag (P4): Requests admitted before the swap are completed under the old header; requests admitted after the swap see the new one. There is no window in which a partially transformed header is servable. If an in-flight request’s user is revoked by the update, the request either completes under the old version (bounded by the admission-to-completion latency, an explicit and tunable exposure window logged for audit) or, under a strict profile, is re-validated against the new version before the partial decryption is released.

## 5. Component Constructions

### 5.1. Verifiable Logging: Permissioned Ledger with On/Off-Chain Storage

We use a permissioned ledger (Hyperledger Fabric [13]) rather than a bare hash chain. Justification: The operators in an outsourcing deployment form a known, closed set, for which a consortium ledger gives finality and throughput without proof of work. Ordering uses a Byzantine-fault-tolerant service [14] when malicious orderers must be tolerated or Raft when only crash faults are expected; the threshold assumption is stated in Section 3. To bound device and storage overhead, only a digest is written on-chain while the event body is stored off-chain under a Merkle commitment (Algorithm 1); the per-event on-chain cost is therefore constant in the payload size.
**Algorithm 1:** Verifiable access-event logging (reference design)  1:**function** LogEvent(user,resource,action,outcome,payload)  2:      body←(user,resource,action,outcome,Now(),H(payload))  3:      cid←OffChainStore(body)           ▹ e.g., content-addressed store  4:      digest←H(body∥cid)  5:      σ←ML-DSA.Sign$\left(sk_{cloud},digest\right)$  6:      Ledger.Append(digest,σ)            ▹ ordered by BFT/Raft service  7:**end function**  8:**function** Verify(digest,σ,body,cid)  9:      **return** ML−DSA. Verify $\left(pk_{cloud},digest,\sigma \right)\wedge digest=H\left(body∥cid\right)$10:**end function**

### 5.2. Decentralized Multi-Authority ABE

We instantiate fine-grained access control with a decentralized MA-CP-ABE scheme [10,11]. Authorities share only global parameters and use a global identifier gid; no inter-authority coordination or central master secret is required, which removes the bottleneck. Algorithm 2 lists the interfaces.
**Algorithm 2:** Decentralized MA-ABE interfaces (reference design)  1:**function** GlobalSetup($1^{\kappa }$) **return** public parameters GP  2:**end function**  3:**function** AuthSetup($\mathrm{GP},{\mathrm{AA}}_{i}$) **return** $\left(pk_{i},sk_{i}\right)$ for the attributes of ${\mathrm{AA}}_{i}$  4:**end function**  5:**function** KeyGen($\mathrm{GP},sk_{i},\mathrm{gid},att$) **return** key share $k_{\mathrm{gid},att}$  6:**end function**  7:**function** Encrypt($\mathrm{GP},\{pk_{i}\},M,A$) **return** CT under access structure A  8:**end function**  9:**function** Decrypt($\mathrm{GP},\{k_{\mathrm{gid},att}\},CT$) **return** *M* if attributes satisfy A10:**end function**

#### 5.2.1. Collusion and Compromise

Collusion resistance follows from binding all of a user’s key shares to gid, so shares from different users do not combine [10]. Compromise of one AA exposes only the attributes it manages: A ciphertext for which its policy also requires an attribute from an honest AA remains protected. The residual risk—loss of availability/integrity for the compromised authority’s attributes—is mitigated by threshold or multi-issuer attribute issuance, which is noted as an extension.

#### 5.2.2. Revocation

We use indirect revocation by attribute-key versioning combined with PRE (Section 5.3): The proxy advances the ciphertext and key version for the affected attribute, so revoked users can no longer decrypt without re-issuing all user keys. A revocation list anchored on-chain provides a verifiable record of revocations.

#### 5.2.3. Verifiable Outsourced Decryption

Because the proxy may be malicious or faulty (Section 3.3), outsourced decryption uses the verifiable variant of Lai, Deng, Guan, and Weng [23]: The ciphertext embeds a commitment that lets the user check, with one cheap local operation, that the partially decrypted token returned by the proxy is correct, and it rejects otherwise. This upgrades outsourced decryption from correctness-by-trust to correctness-by-verification (goal G7) and, together with the signed on-chain event digest, extends verifiability beyond log integrity to the access path itself.

### 5.3. Privacy-Preserving Policy Update via Attribute-Based PRE

On a policy change from A to $A^{\prime }$, the DO derives a re-encryption key $rk_{A\to A^{\prime }}$ and delegates it to the proxy, which transforms the policy-bound components of CT to $CT^{\prime }$ without learning the plaintext (Algorithm 3). Only the policy-dependent ciphertext components and a version tag change, the ASCON-protected symmetric payload is unchanged, which is what makes update cost independent of payload size (principle P1). We instantiate this layer with attribute-based proxy re-encryption in the line initiated by Liang, Cao, Lin, and Shao [24], which realizes exactly the interface of Algorithm 3: A re-encryption key derived from the source and target access structures transforms a CP-ABE ciphertext from policy A to policy $A^{\prime }$ at a semi-trusted proxy that learns nothing about the plaintext. The foundational proxy-cryptography citation [12] is retained only for the origin of the PRE concept, not as the instantiation. We also correct an overclaim in the original submission: Policy privacy against the proxy is an optional extension, not a base guarantee. In the base instantiation, the proxy necessarily learns the target access structure $A^{\prime }$ (it applies it); hiding $A^{\prime }$ as well requires combining AB-PRE with a policy-hiding (anonymous) CP-ABE, which we list as an open integration rather than a claim. Goal G4 is accordingly scoped: plaintext privacy of updates is a base property; policy-hiding is conditional.
**Algorithm 3:** Policy update via attribute-based PRE (reference design)1:**function** ReKeyGen($\mathrm{GP},A,A^{\prime },\mathrm{owner}\;\mathrm{secret}$) **return** $rk_{A\to A^{\prime }}$2:**end function**3:**function** ReEncrypt($CT_{A},rk_{A\to A^{\prime }}$)4:      transform policy-bound components; keep symmetric payload fixed5:    **return** $CT_{A^{\prime }}^{\prime }$          ▹ proxy learns neither *M* nor (if policy-hiding) $A^{\prime }$6:**end function**

### 5.4. Leakage-Resilient Key Handling

We replace the previously unnamed instantiation with a concrete one and scope the guarantee precisely.

Instantiation and λ. Attribute keys are managed under the bounded-leakage model (Definition 1) instantiated by the dual-system leakage-resilient ABE of Lewko, Rouselakis, and Waters [9]. In that construction, a secret key consists of n elements of a group of prime order p and remains secure under leakage of up to $\lambda \approx \left(n-1-2c\right)\log_{2}p$ bits (c a small constant), so the leakage budget is a constant fraction of the key length, and the cost of a larger budget is explicit: Key size and key-side computation grow linearly, $n=\Theta (\lambda /\log_{2}p)$. Integrating this leakage-resilient structure into the decentralized multi-authority setting is not automatic; we state it as composition assumption A2 and invariant I3 in Section Compositional Security: Assumptions, Invariants, and Ideal Functionalities rather than assert it.

Selecting and Revising λ. λ is a deployment parameter, not a constant: it should be set from a side-channel characterization of the actual device class (e.g., measured leakage per trace of the pairing implementation times the number of observable operations per key lifetime, with safety margin), and it must be re-assessed at every hardware or firmware revision.

Leakage Monitoring and Emergency Rotation. The federated monitor’s telemetry includes key-usage counters and timing-anomaly signals, when cumulative estimated leakage for a key approaches a fraction of λ, the architecture triggers the standard revocation path (attribute-version bump plus PRE header update, Section 4.3) as an emergency key rotation, bounding realized leakage per key version.

Session Keys Are Handled by Containment, Not LR-ABE. An ABE leakage-resilience result says nothing about ASCON or ML-KEM-derived session keys, and we no longer imply otherwise. Session keys are protected by short lifetimes (fresh ML-KEM encapsulation per session, so a leaked session key exposes one session), constant-time masked implementations, and key-domain separation (P5). The stronger continual-leakage model for attribute keys remains an option at higher cost.

### 5.5. Layered Lightweight and Post-Quantum Cryptography

The cryptographic stack is layered by function (Table 1): ASCON [20] for on-device authenticated encryption; ML-KEM (FIPS 203, from Kyber [21]) for session-key establishment; ML-DSA (FIPS 204, from Dilithium [22]) for signing ledger entries and policy-update requests; and pairing-based MA-ABE for fine-grained access today, with lattice-based ABE identified as the post-quantum successor. Algorithm 4 sketches the KEM/signature usage. Side-channel resistance relies on constant-time, masked implementations of the lattice primitives.
**Algorithm 4:** PQC usage in the framework (reference design)1:**function** EstablishSession(${\mathrm{pk}}_{\mathrm{kem}}$)2:    (ct,K)←ML−KEM. Encaps $\left({\mathrm{pk}}_{\mathrm{kem}}\right)$; send ct3:    **return** K            ▹ symmetric key for ASCON transport4:**end function**5:**function** SignRequest(${\mathrm{sk}}_{\mathrm{sig}},\mathrm{req}$) **return** ML−DSA.Sign$\left({\mathrm{sk}}_{\mathrm{sig}},\mathrm{req}\right)$6:**end function**

### 5.6. Federated Monitoring Layer

The monitor trains an anomaly detector (an autoencoder or MLP) over decentralized access telemetry without centralizing raw records [15]. To be credible in an adversarial IoT setting, it specifies the following: candidate public datasets (N-BaIoT [25], TON_IoT [26], and Edge-IIoTset [27]); non-IID handling via FedProx [19]; secure aggregation [16] and differential privacy against inference/model-inversion; and Byzantine-robust aggregation (Krum [17], trimmed mean [18]) plus update screening against poisoning and malicious participants. We describe the protocol and its threat handling; we do not report accuracy, which would require the measured study deferred to future work.

## 6. Security Rationale and Compositional Analysis

Following a reviewer’s suggestion, this section is explicitly framed as design rationale: Each item records which component-inherited guarantee supports a goal of Section 3 and under which assumption. These are not system-level theorems; the conditions under which they would compose into system-level guarantees are made explicit in Section Compositional Security: Assumptions, Invariants, and Ideal Functionalities.

**Rationale 1** 
(G1, G2)**.** *Payload confidentiality and collusion resistance are inherited from the IND-CPA security and gid-based collusion resistance of the decentralized MA-ABE scheme [10,11] under its stated assumption. Outsourced decryption preserves security because the proxy receives only a transformation key, not the attribute keys [8].*

**Rationale 2** 
(G3)**.** *Under the bounded-leakage model (Definition 1), the confidentiality of attribute keys persists up to λ bits of leakage per key, inherited from the leakage-resilient ABE of [9]; beyond λ, no guarantee is claimed. This inheritance is conditional on composition assumption A2 and invariant I3 below: The leakage-resilient key structure and the multi-authority key structure must be realized in one compatible construction, which we identify as the principal open construction problem rather than assume is solved. G3 is therefore a design target for which its path is specified, not a property we assert for the composed system.*

**Rationale 3** 
(G4)**.** *Update privacy (plaintext confidentiality of updated ciphertexts) against the honest-but-curious proxy is inherited from the security of attribute-based PRE [24]. Hiding the target policy additionally requires a policy-hiding CP-ABE combined with the AB-PRE, which is scoped as an optional extension (Section 5.3).*

**Rationale 4** 
(G5)**.** *Log integrity and non-repudiation are inherited from collision resistance of H, the BFT threshold of the ordering service [13,14], and EUF-CMA security of ML-DSA [22]: altering a logged event requires a hash collision, a forged signature, or control of more than the tolerated fraction of orderers. These properties are designed to hold even against a malicious cloud (Section 3).*

**Rationale 5** 
(G6)**.** *Transport confidentiality against a harvest-now-decrypt-later adversary is inherited from the IND-CCA security of ML-KEM under module-LWE [21]. We stress that this protects transport and signed artifacts, not the pairing-based ABE layer, which is not post-quantum.*

**Rationale 6** 
(G7)**.** *Detectability of incorrect partial decryption is inherited from the verifiability property of ABE with verifiable outsourced decryption [23]: a proxy that returns a wrong transformation is caught by the user’s local check, except with negligible probability, so a malicious proxy can deny service (which is logged) but cannot cause the silent acceptance of a wrong result.*

### Compositional Security: Assumptions, Invariants, and Ideal Functionalities

Component-wise security does not, in general, imply security of the composition [28]; two reviewers rightly pressed this point. We therefore state explicitly (i) the assumptions under which the above rationales are meaningful, (ii) the invariants that any future composition proof must verify, and (iii) the ideal functionality each module is intended to realize, giving the target specification for a universal composability (UC)-style analysis.

Composition Assumptions. A1 (Key-Domain Separation): No secret is shared across modules, and each module’s keys are generated independently and used only inside that module (principle P5). This removes the most common source of composition failure: cross-module key reuse. A2 (Compatible LR-MA-ABE): There exists a single construction realizing simultaneously the decentralized multi-authority structure of [10,11] and the bounded-leakage resilience of [9]. This is an assumption about a construction, not a theorem; building and proving it is the central open problem this architecture defines. A3 (Interface Confinement): Modules interact only through the messages of Section 4.3; no module exposes side outputs (e.g., the PRE proxy outputs only re-encrypted headers; the ledger outputs only ordered digests).

Invariants to Be Verified by a Future Composition Proof. I1: The ABE-protected header never encapsulates any key from the transport or signing domains (P5 preserved by every workflow step). I2: Every PRE transformation preserves the payload key, which is unchanged, and touches only policy-bound components (P1), so update operations cannot degrade payload confidentiality. I3: Attribute-key leakage bounded by λ does not leak information about the gid-binding randomness that enforces collusion resistance (the coupling point of A2). I4: Version-tag atomicity (P4) holds under concurrent access and update, so no request is ever served from a mixed old/new header. I5: Every access decision, update, and anomaly event is logged before its result is released (log-before-release), so the audit trail is complete with respect to the adversary’s interface.

Ideal Functionalities (Target Specifications). For each module, we state the idealized behaviour a UC-style proof would target: $F_{\mathrm{ABE}}$ releases the payload key exactly to identities for which their attribute set satisfies the current policy version, and the key is released to no one else, tolerating λ-bounded key leakage. $F_{\mathrm{PRE}}$ replaces the policy of a stored header atomically without revealing the payload key to the proxy. $F_{\mathrm{LOG}}$ provides an append-only, totally ordered, non-repudiable record. $F_{\mathrm{KEM}}$ delivers a fresh uniformly random session key to exactly the two endpoints. $F_{\mathrm{SIG}}$ provides the existential unforgeability of policy-update requests and digests. Realizing each functionality from its named construction and proving the composed workflow secure given A1–A3 and I1–I5 are precise statements of the future-work item previously described only informally.

## 7. Analytical Evaluation

We deliberately report no measured performance. Instead, we give standardized parameter sizes and asymptotic complexity, which are public and verifiable, and a qualitative design-space comparison.

Table 1, Table 2 and Table 3 allow a reader to bound the design’s storage/communication footprint and locate it within the design space without any claim that is not independently checkable. The honest comparison in Table 3 also records where the design is not ahead; the access layer is not yet post-quantum.

## 8. Discussion

The architecture embodies explicit trade-offs. On/off-chain storage minimizes device and ledger costs but adds an availability dependency on the off-chain store, mitigated by replication and Merkle verification. PRE-based update trades a one-time re-encryption-key derivation for avoiding full re-encryption, which pays off when policies change more often than the data is rewritten. The leakage-resilient instantiation increases key size in proportion to the leakage bound λ, a direct cost-for-robustness exchange. In the FL layer, Byzantine robustness, differential privacy, and accuracy are in tension: Stronger robustness/privacy generally reduce detection accuracy, and the right operating point is deployment-specific. Finally, the hybrid PQC posture is honest about a gap: transport and signatures are post-quantum, but the pairing-based access layer is not, so the design protects against harvest-now-decrypt-later attacks on transport while leaving long-term ABE confidentiality dependent on the eventual maturity of lattice-based ABE.

## 9. Limitations and Future Work

We state the limitations plainly. (1) No Prototype: This paper presents a design; it reports no measured latency, throughput, energy, or accuracy, and none should be inferred. A prototype with device-level benchmarks and a statistically rigorous evaluation (repeated trials and confidence intervals) is the primary future-work item. (2) Composition Proofs: We provide proof sketches that inherit guarantees from named constructions; the composed system is not proven secure. Section Compositional Security: Assumptions, Invariants, and Ideal Functionalities now makes this limitation actionable by fixing the composition assumptions (A1–A3), the invariants a proof must verify (I1–I5), and per-module ideal functionalities, discharging them, particularly constructing the compatible leakage-resilient multi-authority ABE of assumption A2—remains open; a complete proof for the composed system, ideally machine-checked, remains open. (3) The Access Layer Is Not Post-Quantum: Achieving a fully post-quantum access layer requires maturing and integrating lattice-based ABE. (4) FL Guarantees: Convergence and robustness under severe non-IID and adaptive poisoning are analyzed qualitatively here and must be validated empirically. (5) Parameter Selection: Concrete λ, consensus thresholds, and DP budgets are deployment-specific and require empirical tuning.

## 10. Conclusions

We presented a conceptual reference architecture that composes decentralized MA-ABE, attribute-based proxy re-encryption, permissioned verifiable logging, federated anomaly detection, and lightweight/post-quantum primitives into a single, threat-modeled access-control design for secure IoT outsourcing. Rather than asserting an integrated system and supporting it with simulated numbers, we fixed a system and threat model; instantiated each layer with a named construction; specified the end-to-end data flow; argued security goal-by-goal with explicit caveats; and evaluated the design analytically using only public parameters. The result is a precise, composable, and honestly scoped blueprint for which its intended contribution is to enable—and be tested by—the implementation studies we identify as future work.

## Figures and Tables

**Table 1:** Standardized post-quantum parameter sizes (NIST FIPS 203/204). Values are public constants, not measurements.

Primitive	Role	Public Key	Ciphertext/Sig.	Secret Key
ML-KEM-512	KEM (NIST L1)	800 B	768 B	1632 B
ML-KEM-768	KEM (NIST L3)	1184 B	1088 B	2400 B
ML-KEM-1024	KEM (NIST L5)	1568 B	1568 B	3168 B
ML-DSA-44	Signature (L2)	1312 B	2420 B	2560 B
ML-DSA-65	Signature (L3)	1952 B	3309 B	4032 B
ASCON-128	AEAD	–	+16 B tag	16 B key

**Table 2:** Asymptotic cost of the framework’s operations. |A|: policy size; n: number of authorities; |att|: user attributes.

Operation	Dominant Cost	Notes
Attribute key issuance	O(|att|) per authority	no inter-AA coordination
ABE encryption	O(|A|)	grows with policy size
Outsourced decryption (device)	O(1)	heavy work at proxy
Policy update (PRE)	O(|A|) at proxy	payload untouched
Full re-encryption (baseline)	O(|data|+|A|)	avoided by PRE
On-chain logging	O(1) per event	digest only, body off-chain

**Table 3:** Qualitative design-space comparison (capability presence, not speed).

	Single-Auth	Decentralized	Blockchain	This
	CP-ABE	MA-ABE	ABE	Design
Trust distribution	No	Yes	Partial	Yes
Verifiable audit log	No	No	Yes	Yes
Update without re-enc.	No	No	Varies	Yes (PRE)
Defined leakage model	No	No	No	Yes (λ-bounded)
PQ transport	No	No	No	Yes (ML-KEM)
PQ access layer	No	No	No	No (future: lattice ABE)

## Data Availability

No datasets were generated. The public datasets referenced (N-BaIoT, TON_IoT, and Edge-IIoTset) are available from their respective sources.
